# Supplementary material for: Aequatus: an open-source homology browser
Source: Gigascience. 2018 Nov 5;7(11):giy128. doi: 10.1093/gigascience/giy128 (PMC6251984; doi:10.1093/gigascience/giy128)
Supplement: GIGA-D-18-00226_Revision_1.pdf [file giy128_giga-d-18-00226_revision_1.pdf]

GigaScience

Aequatus: An open-source homology browser

--Manuscript Draft--

**Manuscript Number:**	GIGA-D-18-00226R1
**Full Title:**	Aequatus: An open-source homology browser
**Article Type:**	Technical Note
**Funding Information:**	Biotechnology and Biological Sciences Research Council (GB) (BBS/E/T/000PR5885, BBS/E/T/000PR9817) — Not applicable<br>EU TransPlant (BBS/E/T/000GP006) — Not applicable
**Abstract:**	Background: Phylogenetic information inferred from the study of homologous genes helps us to understand the evolution of genes and gene families, including the identification of ancestral gene duplication events as well as regions under positive or purifying selection within lineages. Gene family and orthogroup characterisation enables the identification of syntenic blocks, which can then be visualised with various tools. Unfortunately, currently available tools display only an overview of syntenic regions as a whole, limited to the gene level, and none provide further details about structural changes within genes, such as the conservation of ancestral exon boundaries amongst multiple genomes.<br>Findings: We present Aequatus, a standalone web-based tool that provides an in-depth view of gene structure across gene families, with various options to render and filter visualisations. It relies on pre-calculated alignment and gene feature information typically held in, but not limited to, the Ensembl Compara and Core databases. We also offer Aequatus.js, a reusable JavaScript module that fulfils the visualisation aspects of Aequatus, available within the Galaxy web platform as a visualisation plugin, which can be used to visualise gene trees generated by the GeneSeqToFamily workflow.<br>Availability: Aequatus is an open-source tool freely available to download under the MIT license at https://github.com/TGAC/Aequatus. A demo server is available at http://aequatus.earlham.ac.uk/. A publicly available instance of the GeneSeqToFamily workflow to generate gene tree information and visualise it using Aequatus is available on the Galaxy EU server at https://usegalaxy.eu.
**Corresponding Author:**	Anil S Thanki, MSc<br>Earlham Institute<br>Norwich, Norfolk UNITED KINGDOM
**Corresponding Author Secondary Information:**	
**Corresponding Author's Institution:**	Earlham Institute
**Corresponding Author's Secondary Institution:**	
**First Author:**	Anil S Thanki, MSc
**First Author Secondary Information:**	
**Order of Authors:**	Anil S Thanki, MSc<br>Nicola Soranzo<br>Javier Herrero<br>Wilfried Haerty<br>Robert P. Davey
**Order of Authors Secondary Information:**	
**Response to Reviewers:**	Reviewer #1: The submission presents a web interface to compare gene structure across different closely related species. Starting with a reference gene, it provides the

Powered by Editorial Manager® and ProduXion Manager® from Aries Systems Corporation

genomic neighbourhood and that of orthologous genes-thus enabling users to visualise local synteny (in a visualisation that is reminiscent of Genomicus). The key innovation, though, lies in a comparative display of gene structure, with an annotated gene tree on the left and a display of matching exons on the right. There are a few additional display options (tables, "sankay plots") but these are not nearly as important. Finally, there is an option to dynamically load information on the domain architecture of any gene.

The widget is well thought out and innovative, and so in general I think it will be quite useful to users. For resource developers, it is a real plus that Aequatus is available both with a full-fledged back-end integrated with Ensembl compara-which is a leading resource-while at the same time available as a JavaScript library only.

Inevitably I have a few points, but these should be easy to address in a revised manuscript.

Major point

1. From a user standpoint, where is or will Aequetus available? For instance, will http://aequatus.earlham.ac.uk/animal_compara/ be kept up to date with the latest Ensembl release? in any case, information about the release should be provided.

A. Yes, we will keep Aequatus available at the given web address. We will try our best to keep Aequatus updated with the latest release of Ensembl. We are also working on a new Aequatus version which will be able to retrieve data directly from Ensembl servers via its API. Moreover, we are going to add the Ensembl release version to the Aequatus webpage to keep the user informed.

2. From a developer standpoint, what is the rough scaling behaviour of the tool in terms of number of genome, number of genes per family etc? What are the bottlenecks? The point is that potential developers will want to know whether the widget can scale to their resource.

A. Aequatus shows the visualisation of a gene family reasonably fast, but since this is generated on the fly, it could behave slower with increasing size and complexity of the gene family (number of paralogues and number of genomes).

Minor points
3. It's not so clear what the colours mean in the gene structure view. I assume these are arbitrary colours that are nevertheless consistent across species, but I still wonder whether there is information in the fact that some exons have different intensities of the same hue, while others don't.

A. As pointed out by the referee the colour were arbitrarily selected to distinguish syntenic genes and matching exons respectively in the syntenic and gene tree views. We have now added a description in the Figure 2 legend.

4. Also unclear to me is the meaning of the tiny arrows between exons, as well as the very fine red, black and white lines. Consider adding a legend.

A. The arrows denote the strand on which the gene is located. We have also updated the Figure 3 legend in the manuscript, adding "In gene tree view, gray blocks at the start and end of each gene represent UTRs (untranslated regions), black bars within exons indicate insertions, red lines represent deletions specific to a given gene compared with the guide, and tiny arrows denotes the coding strand of the gene."

Discretionary/Suggestions:
5. It would be nice to have the possibility to save the current view as SVG, to facilitate inclusion in publications.

A. We appreciate the comment from reviewer about exporting current view as a SVG, this could be very useful. We are looking forward to integrate this into our next release.

Powered by Editorial Manager® and ProduXion Manager® from Aries Systems Corporation

Reviewer #2: Manuscript Title: Aequatus: An open-source homology browser
Manuscript Number: GIGA-D-18-00226
Reviewer: Deborah A Weighill

Summary
This study presents Aequatus, a web-based tool for the visualization of syntenic relationships as well as phylogenetic relationships between genes from pre-calculated alignments and gene features. Aequatus allows for the visualization of gene structure similarities in more detail than other tools, as it can visualize synteny at the exon level.

General Comments
This software will be a useful tool for the visualization of detailed syntenic relationships at the sub-gene level and at the localized gene level, though not at the large, chromosomal scale. The software meets the aims of the study to provide a tool to visualize both phylogenetic and syntenic information and the sub-gene level.

Code for this software is available on github and a demo web server is also available at links provided in the manuscript. The manuscript makes use of data from the Ensembl Compara and Core databases.

The language of this manuscript is of good quality.

Major Comments/Suggestions
1. The demo server appears to have a very limited number of species available for browsing. Is there a plan for this to be expanded so that this can be a browser for the entire Compara database?

A. We indeed plan to expand the set of species to host the Compara databases. The demo server was intended to display Aequatus functionalities, but we are working on mirroring Ensembl Compara locally and then provide Aequatus running on it, serving all available species. We are also working on a new Aequatus version which will be able to retrieve data directly from Ensembl servers via its API to always make all species available at the latest release.

2. The authors list various other tools for synteny visualization and briefly mention how Aequatus differs from these tools. I think it is necessary to expand this discussion/comparison, for example, are these other tools webservers or tools to be run command line? Is there a major speed difference or differences in the size of input data that can be handled? A figure illustrating the output visualizations of these different tools, highlighting how Aequatus differs would be informative.

A. We have added a comparison table along with figures in the Supplementary material to compare the features of various phylogenetic visualisation tools with Aequatus. Regarding the comparison of performance: the performance of each tool is based on the technology used for development, the number of genomes and gene families visualised as well as the complexity of the data. Software performances will also be affected by the configuration of the host server and in some cases the local computer when the visualisation is generated locally. Thus, a performance comparison of various other tools will not enable us to establish a conclusion on which tool performs better.

3. The authors mention in the introduction that Aequatus "allows the identification of exon/intron boundary changes and mutations, informing the user about underlying genetic changes, but can also highlight mis-annotations, pseudogenes [17], or polyploidisation in animal and plant genomes." It is not explicitly illustrated in the manuscript how one can obtain this information using Aequatus. I think that an example (including figures) of how one can arrive at each of these biological insights mentioned in the introduction, using Aequatus, would be very useful. In addition, illustrating how Aequatus facilitates these interpretations better than other tools would be a good point to illustrate explicitly.

Powered by Editorial Manager® and ProduXion Manager® from Aries Systems Corporation

	A. We have moved the cited sentence to the Discussion and clarified it, adding also an example figure where Aequatus is visualising a potential missannotation in the pig genome.<br><br>4. This paper appears to be companion paper to "GeneSeqToFamily: a Galaxy workflow to find gene families based on the Ensembl Compara GeneTrees pipeline" (Anil S Thanki et. al, https://doi.org/10.1093/gigascience/giy005). Citing this paper and explaining more how it fits into the Aequatus pipeline is necessary in my opinion.<br><br>A. We have now added more information about how the Aequatus visualisation tool is working together with the GeneSeqToFamily workflow in Galaxy.<br><br>Minor Comments/Suggestions<br>5. The manuscript abstract says that the software is available for download under an MIT license, whereas on the GitHub page it is distributed under the GNU General Public License. This should be made consistent.<br><br>A. We thank the reviewer for pointing this out. It was a mistake from our side as Aequatus.js is available under MIT license, whereas whole of Aequatus software is GPL v3 license, we have corrected this mistake in manuscript.<br><br>6. Captions for Figures 3 and 4 are missing colons after "Figure".<br><br>A. We have amended manuscript to correct this.
**Additional Information:**	
**Question**	**Response**
Are you submitting this manuscript to a special series or article collection?	No
**Experimental design and statistics**<br><br>Full details of the experimental design and statistical methods used should be given in the Methods section, as detailed in our Minimum Standards Reporting Checklist. Information essential to interpreting the data presented should be made available in the figure legends.<br><br>Have you included all the information requested in your manuscript?	Yes
**Resources**<br><br>A description of all resources used, including antibodies, cell lines, animals and software tools, with enough information to allow them to be uniquely identified, should be included in the Methods section. Authors are strongly encouraged to cite Research Resource Identifiers (RRIDs) for antibodies, model	Yes

organisms and tools, where possible.<br><br>Have you included the information requested as detailed in our Minimum Standards Reporting Checklist?	
**Availability of data and materials**<br><br>All datasets and code on which the conclusions of the paper rely must be either included in your submission or deposited in publicly available repositories (where available and ethically appropriate), referencing such data using a unique identifier in the references and in the “Availability of Data and Materials” section of your manuscript.<br><br>Have you have met the above requirement as detailed in our Minimum Standards Reporting Checklist?	Yes

Manuscript
Click here to access/download;Manuscript;Aequatus .docx
Click here to view linked References

Aequatus: An open-source homology browser

Anil S. Thanki[1, *], Nicola Soranzo[1], Javier Herrero[1,2], Wilfried Haerty[1], Robert P. Davey[1, *]

1. Earlham Institute, Norwich, NR4 7UZ, UK
2. Bill Lyons Informatics Centre, UCL Cancer Institute, London WC1E 6DD, UK

*To whom correspondence should be addressed.

ORCID IDs:

Anil S. Thanki: 0000-0002-8941-444X; Nicola Soranzo: 0000-0003-3627-5340; Javier Herrero: 0000-0001-7313-717X; Wilfried Haerty: 0000-0003-0111-191X; Robert P. Davey: 0000-0002-5589-7754.

Abstract

Background: Phylogenetic information inferred from the study of homologous genes helps us to understand the evolution of genes and gene families, including the identification of ancestral gene duplication events as well as regions under positive or purifying selection within lineages. Gene family and orthogroup characterisation enables the identification of syntenic blocks, which can then be visualised with various tools. Unfortunately, currently available tools display only an overview of syntenic regions as a whole, limited to the gene level, and none provide further details about structural changes within genes, such as the conservation of ancestral exon boundaries amongst multiple genomes.

Findings: We present Aequatus, a standalone web-based tool that provides an in-depth view of gene structure across gene families, with various options to render and filter visualisations. It relies on pre-calculated alignment and gene feature information typically held in, but not limited to, the Ensembl Compara and Core databases. We also offer Aequatus.js, a reusable JavaScript module that fulfils the visualisation aspects of Aequatus, available within the Galaxy web platform as a visualisation plugin, which can be used to visualise gene trees generated by the GeneSeqToFamily workflow.

Availability: Aequatus is an open-source tool freely available to download under the GNU General Public License v3.0 at https://github.com/TGAC/Aequatus. A demo server is available at http://aequatus.earlham.ac.uk/. A publicly available instance of the GeneSeqToFamily workflow to generate gene tree information and visualise it using Aequatus is available on the Galaxy EU server at https://usegalaxy.eu.

Contacts: Anil.Thanki@earlham.ac.uk and Robert.Davey@earlham.ac.uk

Introduction

Sequence conservation across populations or species can be investigated at multiple levels from single nucleotides, to discrete sequences (e.g. transcription factor binding sites, exons, introns), genes, genomic blocks, and chromosomes. Analyses at each of these levels inform different evolutionary processes and time scales. While the vast majority of analyses focus on gene evolution, synteny, (the conservation of genomic blocks between multiple species) can be used to trace chromosome evolutionary history [1] and infer evolutionary relationships between genes across or within species [2]. Synteny resolution and analysis typically involves carrying out multiple sequence alignments (MSAs) and phylogenetic reconstruction, comprising multiple steps that can be computationally intensive even for relatively small numbers of data points [3].

Many methods are available for the identification of genome-wide orthology (MSOAR [4], OrthoMCL [5], OMA [6], HomoloGene [7], PhyOP [8], TreeFam [9], TreeBeST [10]). However, most of them do not incorporate taxonomic information (typically in the form of a species tree) while finding gene families, nor provide any information regarding transcript and protein structural changes across orthogroup members. The Ensembl GeneTrees pipeline [11], a computational workflow developed by the EMBL-EBI Ensembl Compara team, produces familial relationships based on clustering, MSA, and phylogenetic tree inference. The gene trees in Ensembl Compara are inferred with TreeBeST, which relies on a reference species tree to guide the process and calculates the probability of a gene tree in the context of species evolution. The data are stored in a relational database which contains information

on gene families, syntenic regions and protein families. In parallel, the Ensembl Core databases store gene feature information and other genomic annotations at the species level. The Ensembl project (release 90, August 2017) at EMBL-EBI houses 100 vertebrate species [12], along with precomputed MSAs and gene family information.

Phylogenetic reconstruction is the most traditional method to represent and view comparative datasets across a given evolutionary distance, but specific tools such as Ensembl Browser [13], Genomicus [14], SyMAP [15], and MizBee [16] also exist to provide finer-grained information. These tools are able to provide an overview of syntenic regions as a whole, with only Genomicus reaching down to the gene order and orientation level. Conversely, phylogenetic trees retain ancestral information but do not represent the underlying information regarding structural changes within genes, such as the conservation of ancestral exon boundaries between multiple genomes or variants within genes that can be correlated to phenotypic changes. In order to build these gene-level visualisations, basic genomic feature information is required.

Therefore, we have developed Aequatus to bridge the gap between phylogenetic information and gene feature information. Here we show that Aequatus allows the identification of exon/intron boundary changes and mutations, informing the user about underlying genetic changes.

Materials and Methods

Aequatus is built using open-source technologies and is divided into a typical server-client architecture: a web interface and a server backend (see Figure 1).

Figure 1: The Aequatus infrastructure, showing the interactions between the server-side implementation, connected to Ensembl compara and core database using Java Data Access Objects and SMART server via REST API, and the client-side implemented using popular techniques such as JavaScript, jQuery, d3.js and jQuery DataTables.

The server-side component is implemented using the Java programming language. It retrieves and processes comparative genomics information directly from Ensembl Compara

and Ensembl Core databases. Precalculated gene trees and genomic alignments, in the form of CIGAR strings [17], are held in Ensembl Compara, which are cross-referenced by Aequatus to Ensembl Core databases for each species to gather genomic feature information using the unique gene stable IDs.

The Aequatus web interface comprises well-known web technologies such as SVG, jQuery, JavaScript and D3.js [18] to provide a fast and intuitive web-based browsing experience over complex data. Comparative and feature data are processed and rendered in a intuitive graphical interface to provide a visual representation of the phylogenetic and structural relationships among the set of chosen species.

Aequatus visualises gene families using a phylogenetic tree generated from gene sequence conservation information, held in a Ensembl Compara database, and gene features from Ensembl Core database. Gene features are presented in the form of exon-intron boundaries and 5' and 3' UTR. In this gene tree view, users are able to select a gene from a given species as a “guide gene”, and the homologous genes discovered through the comparative analysis are shown with respect to this guide gene. The representation of internal similarity among homologues is achieved by comparing the CIGAR strings for homologous genes with the CIGAR of the guide gene and mapping back to the homologous gene structure.

Aequatus is also able to visualise homologous genes in a customised Sankey view, using the d3.js [18] visualisation library, and provides feature information in an interactive Tabular view, using the jQuery DataTable [19] library. Statistical information for each member in a set of homologues, such as percentage coverage, positivity and identity, are fetched from *homology* and *homology_member* tables of the Ensembl Compara database.

We have integrated a SMART (Simple Modular Architecture Research Tool) [20] service to search for and visualise domain information of a protein sequence. We use the SMART REpresentational State Transfer (REST) API to retrieve protein domains, motifs, signal, repeats information from the SMART server using protein sequences.

Finally, to complement these various visualisations for the homologous genes and their

gene trees, Aequatus provides gene order information in the form of a syntenic view (see Section 3). For a selected gene, homologues are fetched from *homology* and *homology_member* tables of the Ensembl Compara database. The neighbouring genes for these homologous genes are retrieved from the Ensembl Core databases using positional information and organised into a syntenic representation. Much like the shared conserved exon depiction in the gene tree view, syntenic genes are coloured based on the shared homology.

Results

The landing page of Aequatus (see Figure 2) contains a header with a search box (2A) and a dropdown list of species (2B), followed by a selectable Chromosomal view underneath (2C).

Aequatus has a draggable control panel (2G) on the left-hand side, which contains buttons to show/hide the chromosome selector on top, modify gene views and labels, access to the search box and the export options, as well as a link to the help pages.

Figure 2: The main view of Aequatus. The header on top provides a search box (A) and a genome list (B). It is followed by the Chromosomal view (C), where the selected chromosome is coloured in red. Below there is an overview of genes (D) for the selected chromosome, followed by a zoomed area of the chromosome with genes shown in the gene order view (E), and by gene tree view(F). We are using arbitrary colours to distinguish syntenic genes (in gene order view) and matching exons (in gene tree view). The Aequatus control panel (G) is visible on the far left.

1. Aequatus user interface

Aequatus provides various ways to visualise gene trees and the inferred orthology/paralogy from them.

1.1 Main Gene Trees View

The gene tree view (see Figure 3) comprises a phylogenetic tree on the left, built from GeneTree information stored in a Ensembl Compara database [11]. Aequatus relates the genes through different events (e.g duplication, speciation, and gene split) for the gene family and homologous genes against each respective node, which are coloured based on

the potential evolutionary event. Homologous genes are visualised by aligning them against a given guide gene. The selected guide gene is depicted as a larger circle black leaf node in the tree, with a red label on the right, while the other genes have a smaller circle leaf node and a grey label.

On the right, Aequatus depicts the internal gene structure, using a shared colour scheme for coding regions, to represent similarity across homologues. Homologous genes are visualised by aligning them against a given guide gene. Aequatus is also able to indicate insertions and deletions in homologous genes with respect to shared ancestors. Black bars within exons represent insertions, while red lines represent deletions specific to a given gene compared with the guide.

Aequatus provides two view types for gene families. The first (default) view is exon-focused (as in Figure 3), where all introns are set to a fixed width, since long introns can adversely affect the visibility of surrounding exons. This provides easier browsing of the actual gene structure, especially when less screen real estate is available. Conversely, in the second view all homologous genes are resized to the maximum available width in the web browser, showing introns and exons proportional to the real gene size. Users can switch between these views from the "Introns" settings in the control panel.

Figure 3: The genetree for the monoamine oxidase (MAO) gene, with the Chimp gene as the reference, alongside other homologous genes in the exon-focused view. Considering the gene tree on the left, it is clear that the MAO genes are separated into two clusters, corresponding to the MAO-A and MAO-B gene families.

In gene tree view, gray blocks at the start and end of each gene represent UTRs (untranslated regions), black bars within exons indicate insertions, red lines represent deletions specific to a given gene compared with the guide, and tiny arrows denotes the coding strand of the gene.

1.1.1 Popups

Aequatus provides a contextual menu system via interactive popup menus, which are

displayed when a user clicks on a gene (see Figure 4). Each popup shows: the gene name and its position; a link to find protein domain information using SMART; links to export the protein sequence or the CIGAR alignment; an option to set the current gene as the guide in order to see insertions and deletions in homologous genes relative to the selected guide gene; a link out to the Ensembl page for the gene; an option to view the pairwise alignment.

Figure 4: The popup in the gene tree view when clicking on a gene. The popup contains the chromosome name and position, and options to view the protein domains, export the sequence or the alignment, change the guide gene, connect to the Ensembl page for the gene, and view the pairwise alignment.

1.1.2 Protein Domain

Aequatus can provide an interactive visualisation of the protein domains for the selected gene. Aequatus finds the protein domains by connecting to the SMART web server via its REST API and querying the protein sequence for domains, motifs, internal repeats, etc. In this view (see Figure 5), a user can filter and sort domains based on type, E-value, position and source of domain. The features shown in the diagram can be exported in CSV (comma-separated values) or Excel file format.

Figure 5: Visualisation of the protein domain information for the protein ENSPTRP00000037440 retrieved from the SMART server. On the top, drawings of domains mapped on exons (shown with red lines). The tables below are listing the features shown in the diagram, as well as hidden features.

1.2 Homologous Genes

The underlying information describing homologous genes contained within the Compara database schema can be visualised using either a tabular view or Sankey plot.

1.2.1 Tabular View

The Tabular view (see Figure 6) contains statistical information for the homologous relationships. This view is dynamic, allowing the user to search for any homolog using a search box (6A) as well as filter results for the type of homology (6E) (1-to-1 orthologs, 1-to-many orthologs, and paralogs) or one or more specified species (6D). Homologous genes can be exported from the tabular view as Excel, CSV or PDF.

Figure 6: Homologous for the gene MAOA (ENSPTRG00000021816) in tabular view with statistical comparison about homologues. The tabular view contains a search box on top (A). There are 2 buttons to visualise statistical comparisons (C) and pairwise alignment (D) for each homolog. At the bottom it is possible to select from a list of species (D) and the type of homology (E).

Extra details for the pairwise alignment between homologues can be shown by using the '+' button for the homologue entry. The first button (6B) will show statistical comparisons for identity, coverage, similarity etc., while the second button (6C) will visualise the pairwise alignment with the gene structures as detailed below (Figure 8B).

1.2.2 Sankey view

The Sankey view (see Figure 7) visualises homology as a interactive diagram, where the homologues of a selected gene are distinguished by homology type, i.e. paralogs, 1-to-1 orthologs, or 1-to-many orthologs. The nodes for homologous genes are coloured by species, which helps finding genes from the same species in the case of 1-to-many and many-to-many orthologs.

When clicking on a homologous gene, additional details for the homologous pair are displayed in the info panel on the right-hand side.

Figure 7: Homologues for a gene in Sankey format, grouped together by type of homology. The control panel on the left shows filters for the view. Further information for any homologue can be retrieved by clicking on it, which are then shown in a box on the right.

2. 1-to-1 alignment

1-to-1 alignments between homologous genes are important for pairwise comparison. 1-to-1 alignments (Figure 8) can be seen by clicking on the corresponding option either in the popup for the gene tree view or in the homologous genes tabular view. This will fetch the relevant alignment from the homology table of the Ensembl Compara database and visualise it based on the gene structure (8A), together with the pairwise protein sequence alignments (8B).

Figure 8: 1-to-1 alignments between homologous genes. On the top (A) visualising alignment on gene structure and on the bottom (B) visualising pairwise sequence alignments.

3. Gene Order

Genes that share a common ancestor and are part of a consecutive block of genes are likely to have a transcriptional and/or functional relationship [21]. Hence, inferred homologues which are present in all species and in the same order are more likely to be real homologues. In the Gene Order view, neighbouring genes are displayed for the selected gene and its homologues (shown in Figure 9). Homologues of the genes in neighbouring species are coloured based on the matching genes from the reference species. Clicking on a gene feature will open a search panel with various viewing options, and mousing over a given gene will highlight all homologous genes within the same region. The syntenic view complements the main functionality of Aequatus by providing evidence for the conservation information for the genes of interest.

Figure 9: Gene Order for the MAOA gene in Pan troglodytes, where they are coloured by homologous genes. The selected gene and its homologous have a red border. White genes are the ones which don't have any homologous in current visible region.

4. Search

Aequatus has keyword-based search functionality, whereby the user can provide search terms and a list of all the relevant genes is returned. Aequatus can query for matching gene symbols, Ensembl stable IDs (unique identifiers in the Ensembl project for each genomic annotation), common names for genes and proteins, or any keyword in the description. Search results then allow the user to visualise the corresponding gene tree view, or homologous genes in the tabular or Sankey views.

5. Export

Users can export data at different points in the visualisation. In the gene tree view the underlying genomic data for the gene families can be exported in various forms, such as a list of gene IDs, the sequence alignments, or the gene trees in Newick [22] or JavaScript Object Notation (JSON) [23] format, for use in downstream tools. The tabular view can be exported in CSV, XLS, and PDF format.

6. Persistent URLs

Aequatus provides persistent unique URLs to enable consistent access to genes of interest,

making it easy to go back to the results of a previous search, to share information with collaborators, or for use in publications. Users can share the link for the visualisation of a specific gene, the results of a search for a term, or a specific reference to a given species and chromosome.

Discussion

The ultimate goal of Aequatus is to provide a unique and informative way to render and explore complex relationships between genes from various species at a level of detail that has so far been unrealised in a single platform. Table 1 in Supplementary material shows a detailed comparison of Aequatus with various phylogenetic visualisation tools, which highlights the signature feature of Aequatus, i.e. genetic structural comparison. Figures 1-3 of the Supplementary material allow a comparison of the visualisations of MAOB genes from the tools offering a gene tree-focused view.

While applicable to species with high-quality gold-standard reference genomes present in core database resources such as human or mouse, Aequatus has been designed to accommodate users that need to explore large, fragmented, non-model genome references that are held in institutional databases. Comparing non-model organism genes with gold standard genomes allows the identification of exon/intron boundary changes and mutations, informing the user about underlying genetic changes, but can also highlight mis-annotations, pseudogenes [24], or polyploidisation (see Figure 10). We are currently testing Aequatus with a range of non-model organisms, such as koala, polyploid crops, and spiny mouse. As Aequatus can visualise relationships using simple CIGAR strings, any tool that outputs this format can use Aequatus to view them. We produce input for Aequatus using the GeneSeqToFamily pipeline, a freely available Galaxy workflow [25] for finding and visualising gene families for genomes which are not available from Ensembl databases.

Figure 10: The gene tree view for the insulin receptor (INSR) gene, with the chimp gene as the guide alongside other homologous genes. "B" and "C" point to two genes from the pig genome, which are matching two different parts of the guide gene (shown with dotted rectangles in corresponding colors). "A" instead indicates an exon of one of the pig genes (enlarged in "D") matching 2 adjacent exons of the guide gene. All these may

suggest a potential gene split event or just a mis-annotation.

In order to make Aequatus more accessible and reusable, the gene tree visualisation module from the standalone Aequatus browser is available as Aequatus.js [26], an open source JavaScript library. In this way, it preserves the interactive functionality of the Aequatus browser tool but can be integrated with other third-party web applications. We have demonstrated this by integrating the Aequatus.js library into Galaxy [27], where gene families generated by running the GeneSeqToFamily workflow can be visualised using the Aequatus plugin within Galaxy.

Future Directions

The main extension to the functionalities of Aequatus is the incorporation of Ensembl REST API functionality [28], where Aequatus will be able to retrieve information directly from Ensembl Compara and Core databases held at the EMBL-EBI, without any need for local database configuration. Whilst this will mean that users will need a reliable internet connection, it will reduce the need for local storage space for the Core databases, improving the portability of Aequatus.

We also intend to containerise Aequatus using Docker and CyVerse UK [29], and BioConda [30] with Galaxy [25,27]. We will produce new APIs between Aequatus and TGAC Browser [31] to provide a comprehensive solution for genome analysis and exploration focused on non-model organisms.

Availability of supporting source code and requirements

- Project name: Aequatus: Earlham Institute's Synteny Browser
- Project home page: https://github.com/TGAC/Aequatus
- Operating systems: Platform independent
- Programming language: Java
- Other requirements: Java 1.7, Maven 2.0, Apache Tomcat, Ensembl Compara and core MySQL databases.

- License: GNU GPL v3.

Availability of Supporting Data

Snapshots of the code are available from the *GigaScience* GigaDB database [32].

Abbreviations

API: Application Programming Interface; CSV: comma-separated values file; JSON: JavaScript Object Notation; MSA: multiple sequence alignment; REST: REpresentational State Transfer; SMART: Simple Modular Architecture Research Tool; URL: Uniform Resource Locator; UTR: untranslated region.

Acknowledgements

This work was strategically funded by the BBSRC (BBS/E/T/000PR5885, BBS/E/T/000PR9817) and through the EU TransPlant grant (BBS/E/T/000GP006). GeneSeqToFamily and the EI Galaxy platform are funded through the BBSRC-supported EI National Capability in e-Infrastructure (BBS/E/T/000PR9814).

This work was supported in part by the NBI Computing Infrastructure for Science Group, which provides technical support and maintenance to EI's high-performance computing cluster and storage systems, which enabled us to develop this tool.

Conflict of Interest: none declared.

References

1. Synteny: Inferring Ancestral Genomes | Learn Science at Scitable. https://www.nature.com/scitable/topicpage/synteny-inferring-ancestral-genomes-44022

2. Vilella AJ, Severin J, Ureta-Vidal A, Heng L, Durbin R, Birney E. EnsemblCompara GeneTrees: Complete, duplication-aware phylogenetic trees in vertebrates. Genome Res. 2009;19:327–35.

3. Edgar RC, Batzoglou S. Multiple sequence alignment. Curr Opin Struct Biol. 2006;16:368–73.

4. Fu Z, Chen X, Vacic V, Nan P, Zhong Y, Jiang T. MSOAR: a high-throughput ortholog assignment system based on genome rearrangement. J Comput Biol. 2007;14:1160–75.

5. Li L, Stoeckert CJ Jr, Roos DS. OrthoMCL: identification of ortholog groups for eukaryotic genomes. Genome Res. 2003;13:2178–89.

6. Altenhoff AM, Dessimoz C. Inferring Orthology and Paralogy. Methods in Molecular Biology. 2012. p. 259–79.

7. Wheeler DL, Barrett T, Benson DA, Bryant SH, Canese K, Chetvernin V, et al. Database resources of the National Center for Biotechnology Information. Nucleic Acids Res. 2008;36:D13–21.

8. Goodstadt L, Ponting CP. Phylogenetic reconstruction of orthology, paralogy, and conserved synteny for dog and human. PLoS Comput Biol. 2006;2:e133.

9. Li H, Coghlan A, Ruan J, Coin LJ, Hériché J-K, Osmotherly L, et al. TreeFam: a curated database of phylogenetic trees of animal gene families. Nucleic Acids Res. 2006;34:D572–80.

10. TreeSoft: TreeBeST. http://treesoft.sourceforge.net/treebest.shtml Accessed 9th June 2018

11. Clamp M, Andrews D, Barker D, Bevan P, Cameron G, Chen Y, et al. Ensembl 2002: accommodating comparative genomics. Nucleic Acids Res. 2003;31:38–42.

12. Zerbino DR, Achuthan P, Akanni W, Amode MR, Barrell D, Bhai J, et al. Ensembl 2018. Nucleic Acids Res. Oxford University Press; 2018;46:D754–61.

13. Stalker J, Gibbins B, Meidl P, Smith J, Spooner W, Hotz H-R, et al. The Ensembl Web site: mechanics of a genome browser. Genome Res. 2004;14:951–5.

14. Muffato M, Louis A, Poisnel CE, Crollius HR. Genomicus: a database and a browser to study gene synteny in modern and ancestral genomes. Bioinformatics. 2010;26:1119–21.

15. Soderlund C, Nelson W, Shoemaker A, Paterson A. SyMAP: A system for discovering and viewing syntenic regions of FPC maps. Genome Res. 2006;16:1159–68.

16. Meyer M, Munzner T, Pfister H. MizBee: a multiscale synteny browser. IEEE Trans Vis Comput Graph. 2009;15:897–904.

17. Sequence Alignment/Map Format Specification. http://samtools.github.io/hts-specs/SAMv1.pdf Accessed 9th June 2018

18. Bostock M. D3.js - Data-Driven Documents. http://d3js.org/ Accessed 9th June 2018

19. DataTables | Table plug-in for jQuery. https://datatables.net. Accessed 9th June 2018

20. Schultz J, Milpetz F, Bork P, Ponting CP. SMART, a simple modular architecture research tool: Identification of signaling domains. Proceedings of the National Academy of Sciences. 1998;95:5857–64.

21. Dávila López M E al. Analysis of gene order conservation in eukaryotes identifies transcriptionally and functionally linked genes. PLoS One. 2010 May 14;5(5):e10654. doi: 10.1371/journal.pone.0010654.

22. Newick Format. http://evolution.genetics.washington.edu/phylip/newick_doc.html. Accessed 8 Apr 2018.

23. JSON [Internet]. [cited 2017 Aug 16]. Available from: http://www.json.org

24. Vanin EF. Processed pseudogenes: characteristics and evolution. Annu Rev Genet. 1985;19:253–72.

25. Thanki AS, Soranzo N, Haerty W, Davey RP. GeneSeqToFamily: a Galaxy workflow to find gene families based on the Ensembl Compara GeneTrees pipeline. Gigascience. 2018 Mar 1;7(3):1-10. doi:10.1093/gigascience/giy005.

26. Thanki AS, Davey RP. TGAC/aequatus.js GitHub Repository. https://github.com/TGAC/aequatus.js

27. Afgan E, Baker D, van den Beek M, Blankenberg D, Bouvier D, Čech M, et al. The Galaxy platform for accessible, reproducible and collaborative biomedical analyses: 2016 update. Nucleic Acids Res. Oxford University Press; 2016;44:W3–10.

28. Yates A, Beal K, Keenan S, McLaren W, Pignatelli M, Ritchie GRS, et al. The Ensembl REST API: Ensembl Data for Any Language. Bioinformatics. 2015;31:143–5.

29. Goff SA, Vaughn M, McKay S, Lyons E, Stapleton AE, Gessler D, et al. The iPlant Collaborative: Cyberinfrastructure for Plant Biology. Front Plant Sci. 2011;2:34.

30. Grüning B, Dale R, Sjödin A, Chapman BA, Rowe J, Tomkins-Tinch CH, Valieris R, Köster J; Bioconda Team. Bioconda: sustainable and comprehensive software distribution for the life sciences. Nat Methods. 2018 Jul;15(7):475-476. doi: 10.1038/s41592-018-0046-7.

31. Anil S Thanki, Xingdong Bian, Robert P Davey. TGAC Browser: visualisation solutions for big data in the genomic era. http://browser.earlham.ac.uk/ Accessed 8th June 2018.

32. Thanki AS; Soranzo N; Herrero J; Haerty W; Davey RP (2018): Supporting data for "Aequatus: An open-source homology browser" GigaScience Database. http://dx.doi.org/10.5524/100509

Click here to access/download;Figure;Figure1.png

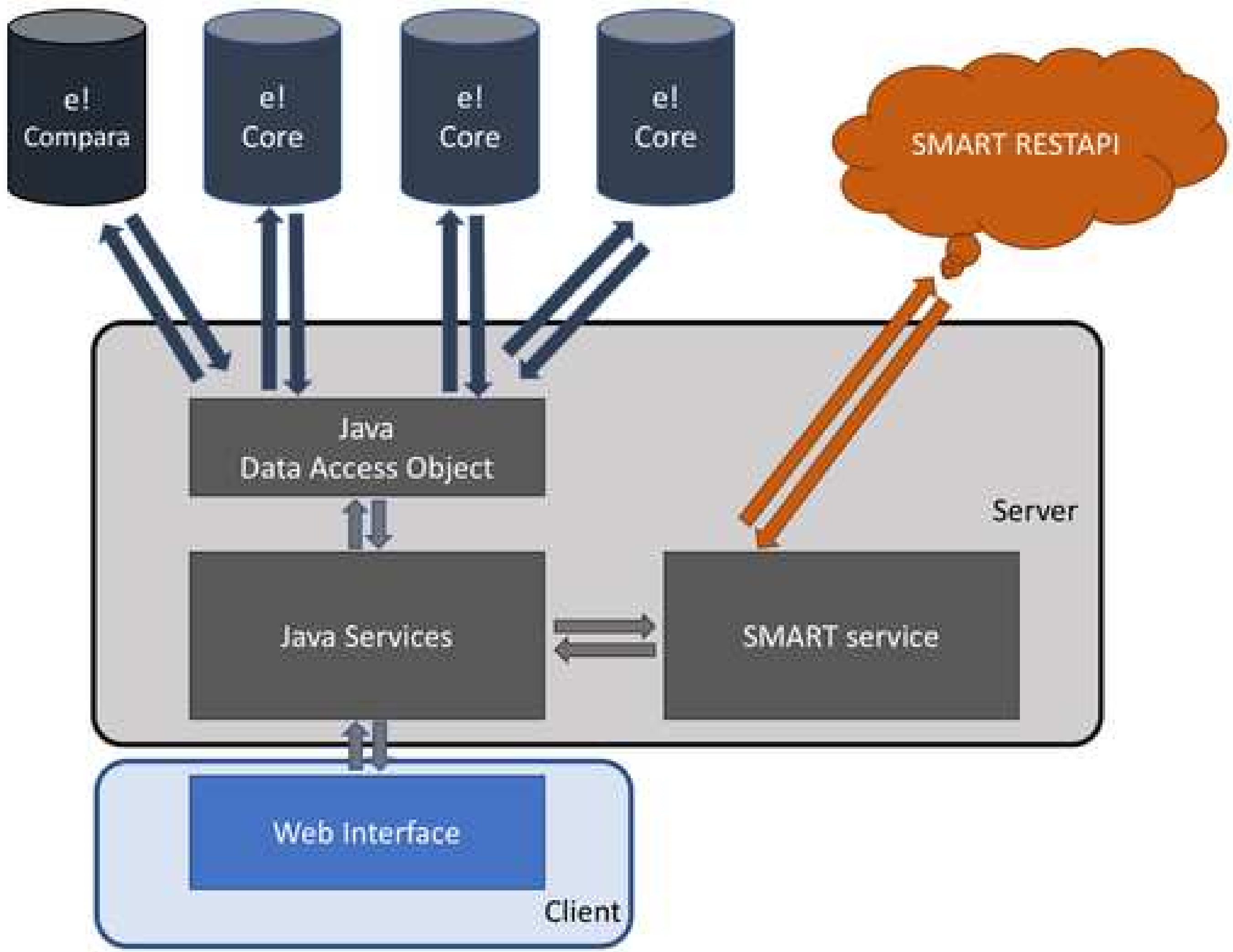

Click here to access/download;Figure;Figure2.png

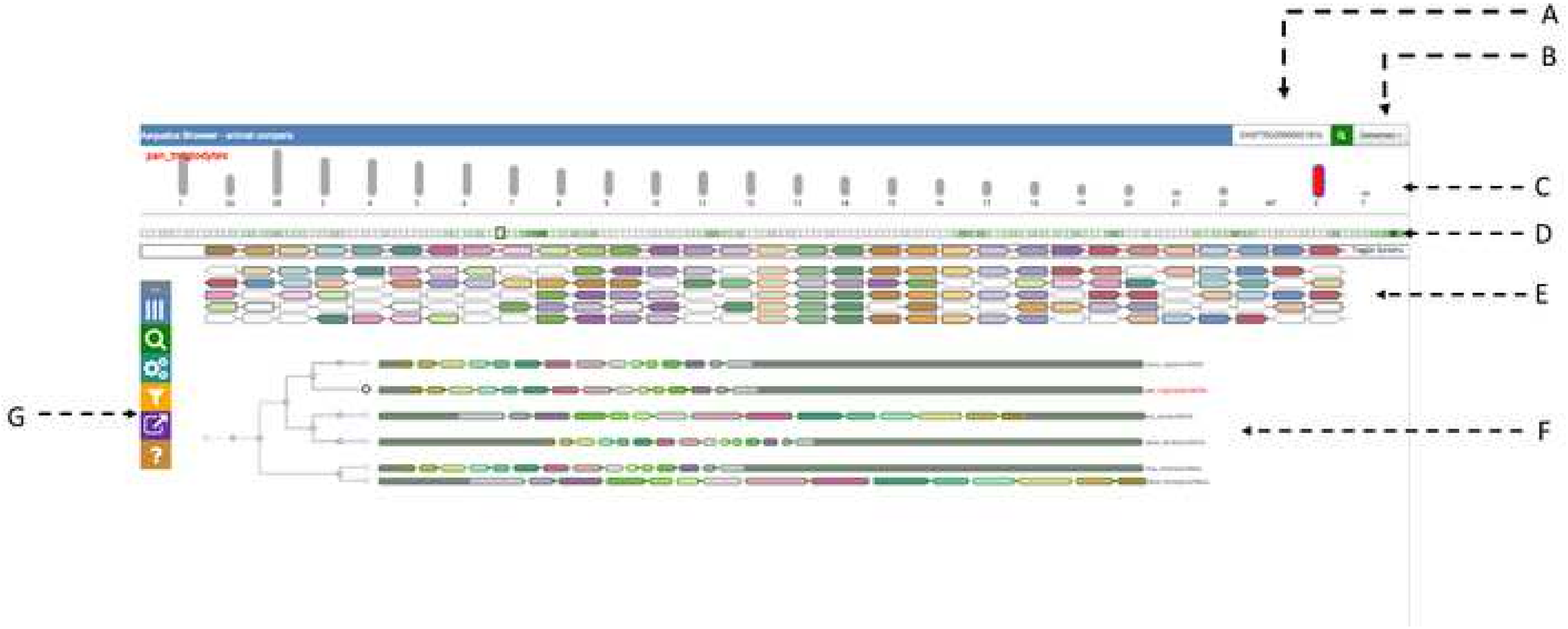

Click here to access/download;Figure;Figure3.png

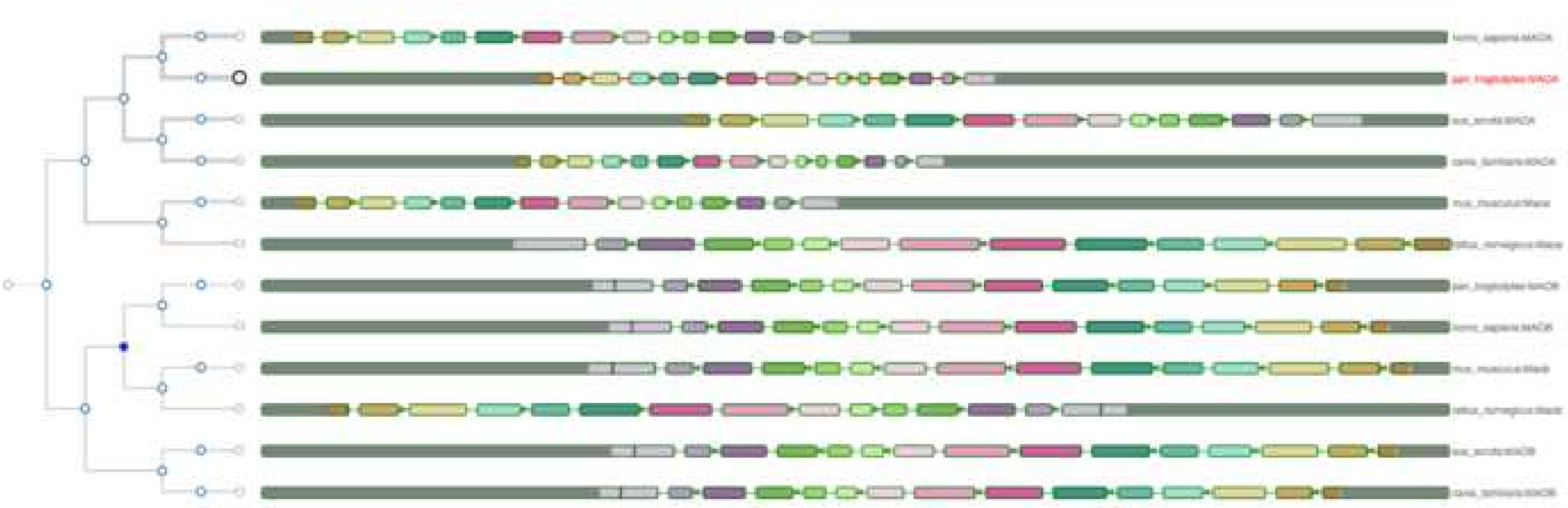

Click here to access/download;Figure;Figure4.png

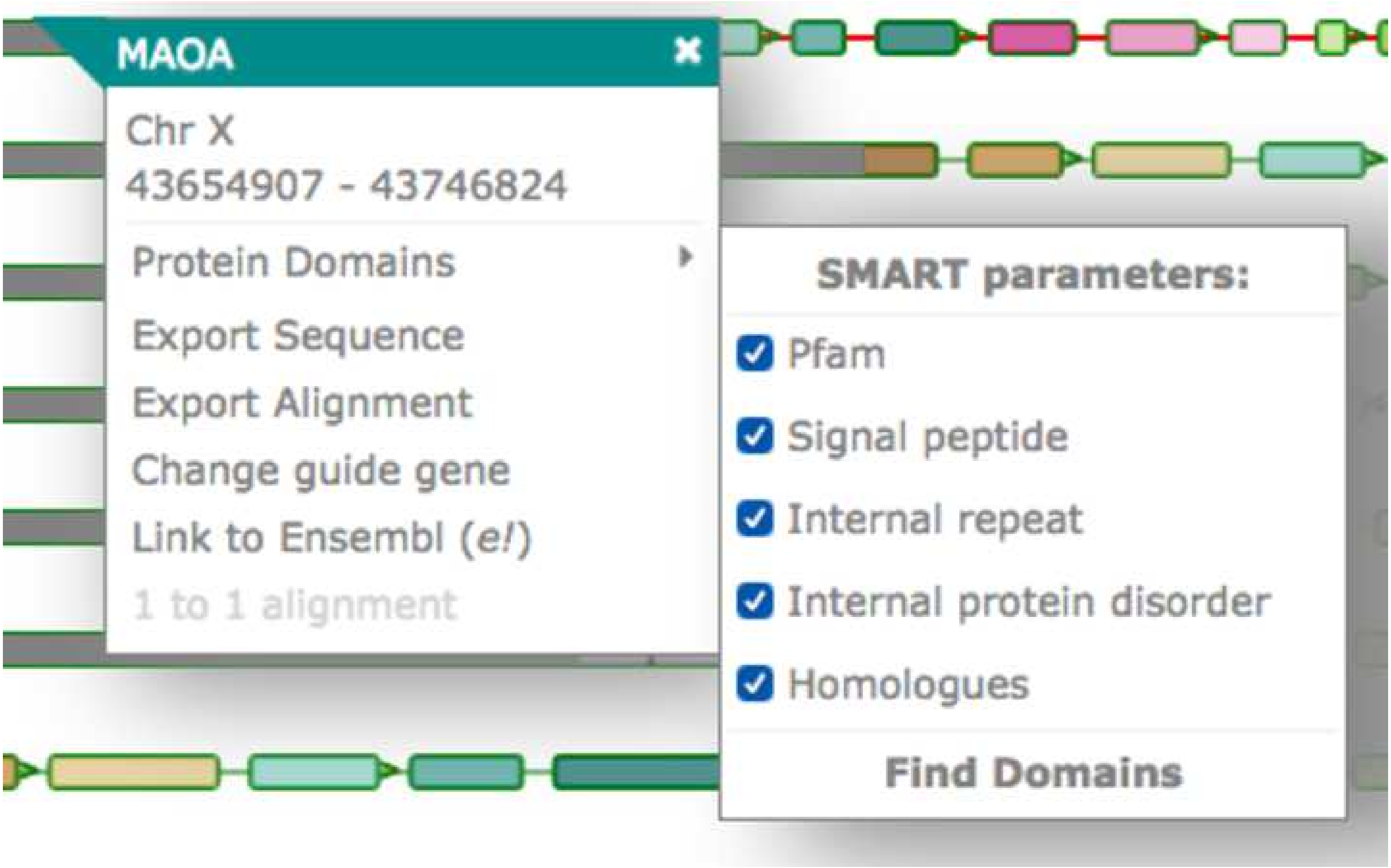

Click here to access/download;Figure;Figure5.png

Protein Domains

Domains within pan_troglodytes protein ENSPTRP00000037440

Legends: PFAM domains SMART domains Low Complexity Region Internal Repeats Signal Peptide Other Intrinsic Region

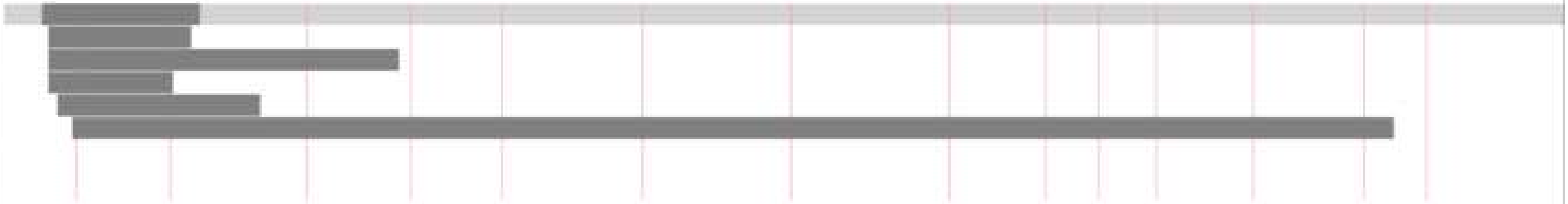

Confidently predicted domains, repeats, motifs and features from SMART

CSV Search:

Name	Start	End	E-value	Type
Pfam:FAD_binding_3	13	64	1.5e-09	PFAM
Pfam:Pyr_redox	15	61	1.2e-07	PFAM
Pfam:DAO	15	130	1.3e-09	PFAM
Pfam:FAD_binding_2	15	55	1.9e-07	PFAM
Pfam:NAD_binding_8	18	84	1.2e-16	PFAM
Pfam:Amino_oxidase	23	460	2.6e-76	PFAM
Name	Start	End	Select E-value	Select Type

Showing 1 to 6 of 6 entries Previous 1 Next

Features NOT shown in the diagram:

Show 10 entries Search:

Name	Start	End	E-value	Type	Reason
PDB:2BXS:B	1	527	0.0	PDB	overlap
SCOP:d1f8ra1	13	338	3e-49	SMART	overlap
low_complexity_region	16	23	0	INTRINSIC	overlap
low_complexity_region	60	71	0	INTRINSIC	overlap
low_complexity_region	446	459	0	INTRINSIC	overlap
Name	Start	End	E-value	Type	Reason

Showing 1 to 5 of 5 entries Previous 1 Nex

Click here to access/download;Figure;Figure6.png

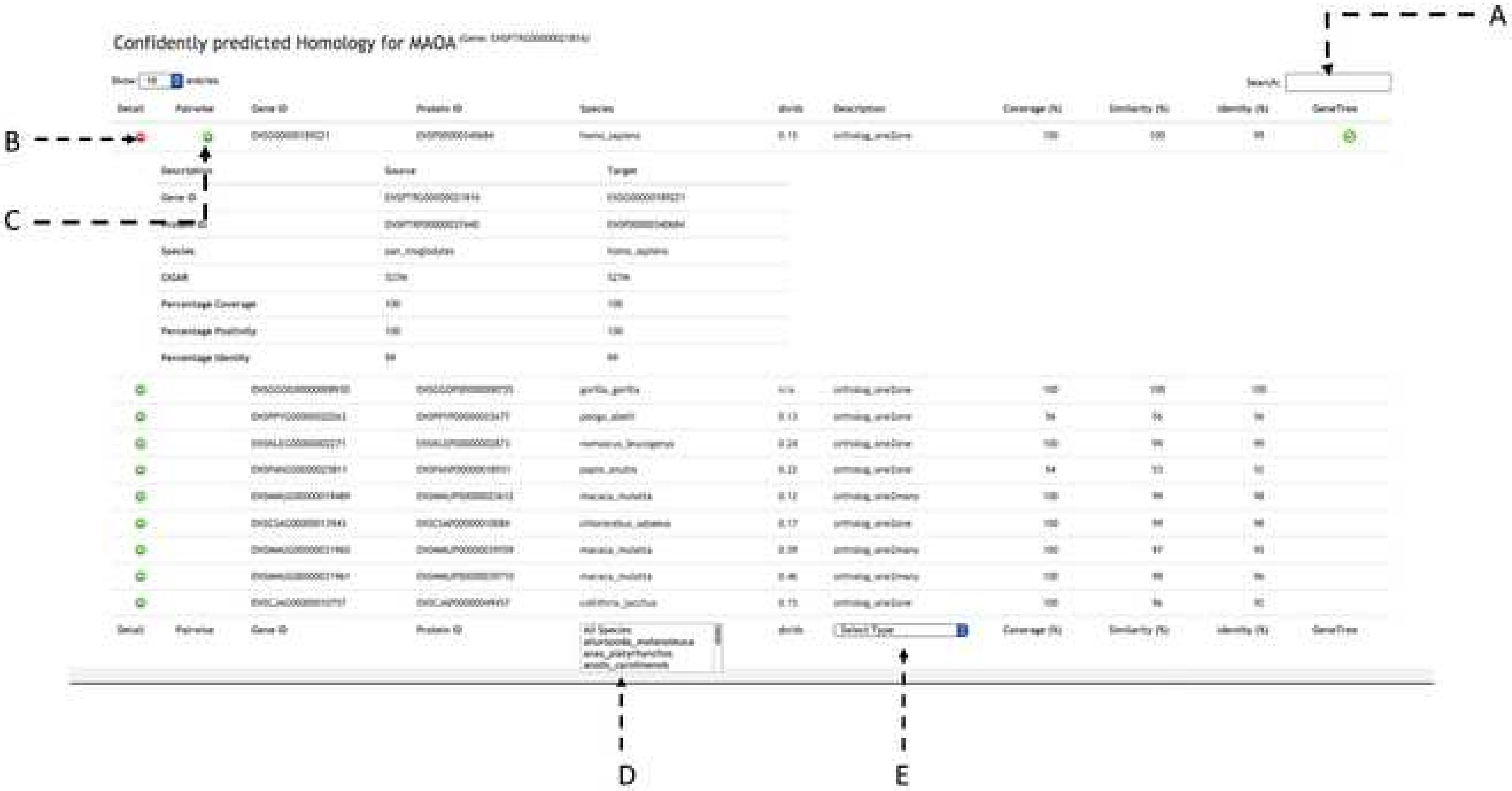

Click here to access/download;Figure;Figure7.png

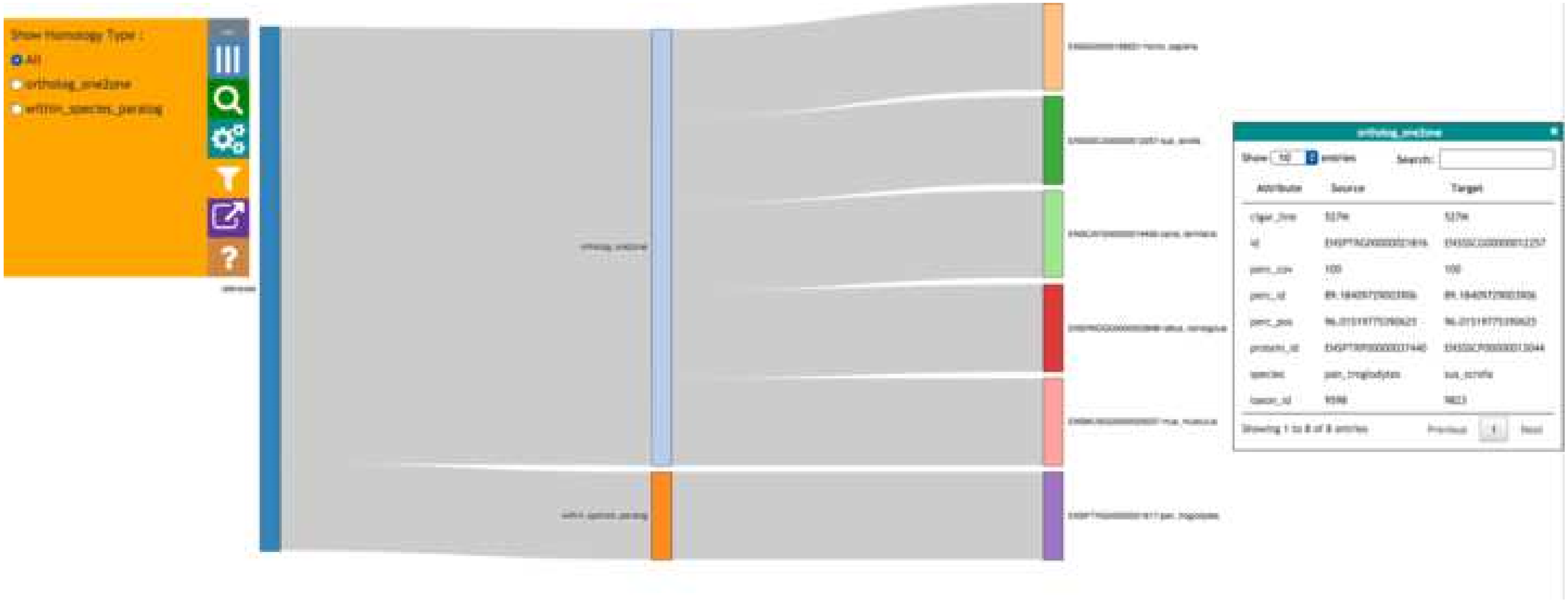

Click here to access/download;Figure;Figure8.png

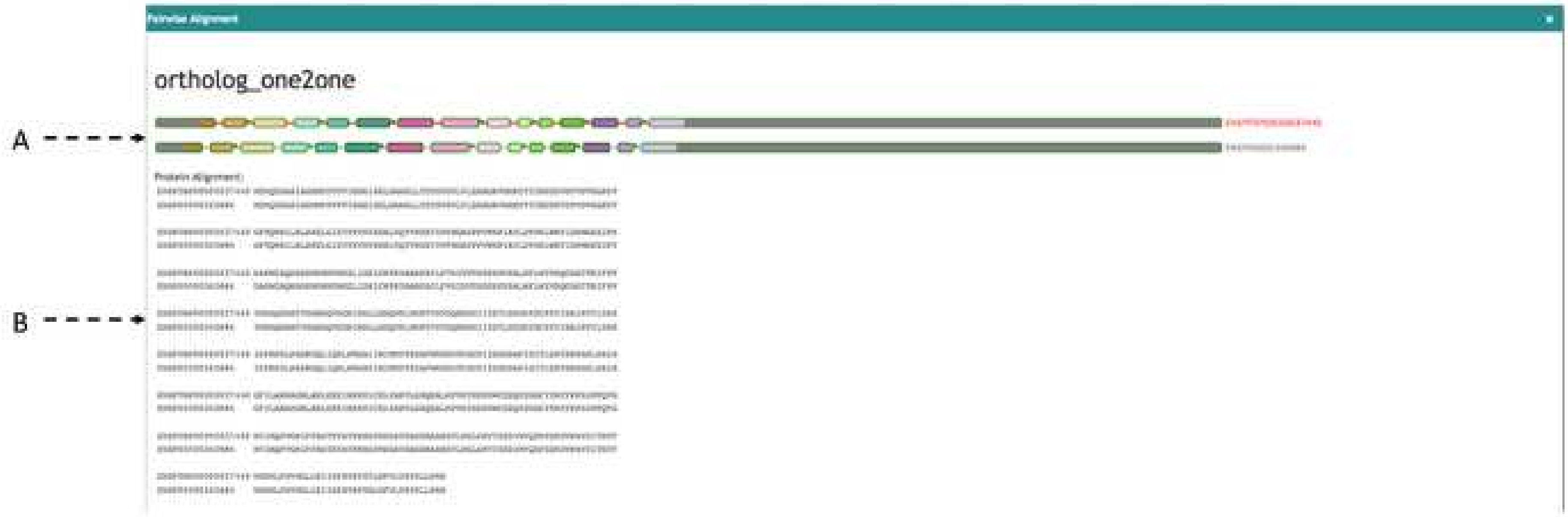

Click here to access/download;Figure;Figure9.png

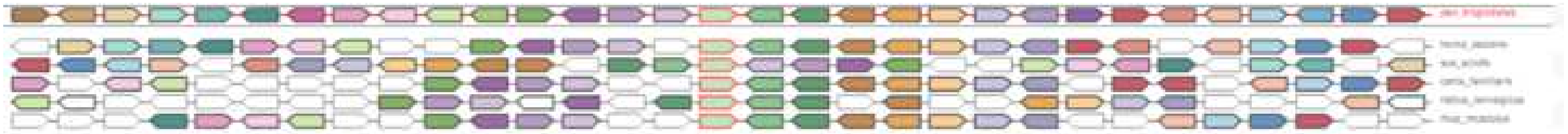

Click here to access/download;Figure;Figure10.png

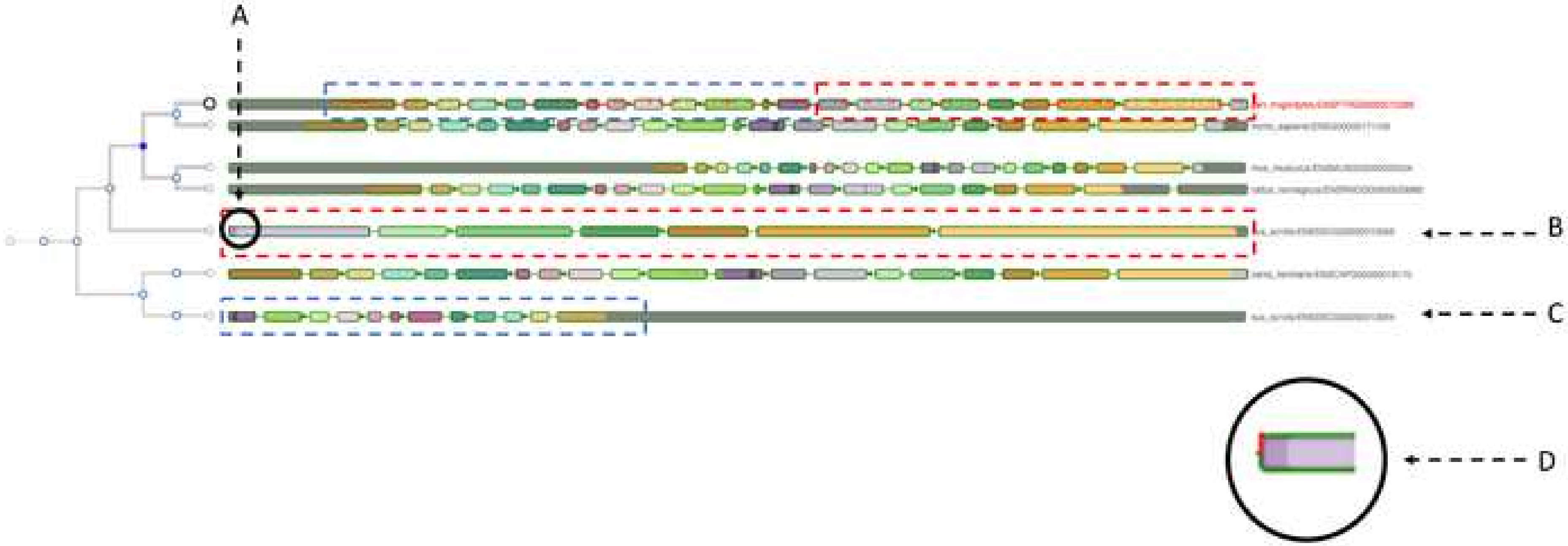

Click here to access/download
Supplementary Material
Aequatus - supplimentary material.docx
