# Supplementary material for: Aequatus: an open-source homology browser
Source: Gigascience. 2018 Nov 5;7(11):giy128. doi: 10.1093/gigascience/giy128 (PMC6251984; doi:10.1093/gigascience/giy128)
Supplement: Reviewer_2_Original_Submission_Attachment.pdf [file giy128_reviewer_2_original_submission_attachment.pdf]

Manuscript Title: Aequatus: An open-source homology browser
Manuscript Number: GIGA-D-18-00226
Reviewer: Deborah A Weighill

Summary
This study presents Aequatus, a web-based tool for the visualization of syntenic relationships as well as phylogenetic relationships between genes from pre-calculated alignments and gene features. Aequatus allows for the visualization of gene structure similarities in more detail than other tools, as it can visualize synteny at the exon level.

General Comments
This software will be a useful tool for the visualization of detailed syntenic relationships at the sub-gene level and at the localized gene level, though not at the large, chromosomal scale. The software meets the aims of the study to provide a tool to visualize both phylogenetic and syntenic information and the sub-gene level.

Code for this software is available on github and a demo web server is also available at links provided in the manuscript. The manuscript makes use of data from the Ensembl Compara and Core databases.

The language of this manuscript is of good quality.

Major Comments/Suggestions

1. The demo server appears to have a very limited number of species available for browsing. Is there a plan for this to be expanded so that this can be a browser for the entire Compara database?
2. The authors list various other tools for synteny visualization and briefly mention how Aequatus differs from these tools. I think it is necessary to expand this discussion/comparison, for example, are these other tools webservers or tools to be run command line? Is there a major speed difference or differences in the size of input data that can be handled? A figure illustrating the output visualizations of these different tools, highlighting how Aequatus differs would be informative.
3. The authors mention in the introduction that Aequatus *"allows the identification of exon/intron boundary changes and mutations, informing the user about underlying genetic changes, but can also highlight mis-annotations, pseudogenes [17], or polyploidisation in animal and plant genomes."* It is not explicitly illustrated in the manuscript how one can obtain this information using Aequatus. I think that an example (including figures) of how one can arrive at each of these biological insights mentioned in the introduction, using Aequatus, would be very useful. In addition, illustrating how Aequatus facilitates these interpretations better than other tools would be a good point to illustrate explicitly.
4. This paper appears to be companion paper to "GeneSeqToFamily: a Galaxy workflow to find gene families based on the Ensembl Compara GeneTrees pipeline" (Anil S Thanki et. al, https://doi.org/10.1093/gigascience/giy005). Citing this paper and explaining more how it fits into the Aequatus pipeline is necessary in my opinion.

Minor Comments/Suggestions

1. The manuscript abstract says that the software is available for download under an MIT license, whereas on the github page it is distributed under the GNU General Public License. This should be made consistent.
2. Captions for Figures 3 and 4 are missing colons after "Figure".

Recommendations

I think that this is a useful tool and that the manuscript should be published after addressing the suggested additional discussions above, particularly providing some more discussion of examples of biological conclusions to be drawn from such analyses, and a further discussion of the differences between this tool and other syntenic/phylogenetic visualization tools.
