# Supplementary material for: Aequatus: an open-source homology browser
Source: Gigascience. 2018 Nov 5;7(11):giy128. doi: 10.1093/gigascience/giy128 (PMC6251984; doi:10.1093/gigascience/giy128)
Supplement: Supplemental File [file giy128_supplemental_file.docx]

**Aequatus: An open-source homology browser**

Anil S. Thanki^1, *^, Nicola Soranzo^1^, Javier Herrero^1,2^, Wilfried Haerty^1^, Robert P. Davey^1, *^

1. Earlham Institute, Norwich, NR4 7UZ, UK
2. Bill Lyons Informatics Centre, UCL Cancer Institute, London WC1E 6DD, UK

*To whom correspondence should be addressed.

# Supplementary file

| **Feature** | **Aequatus** | **Ensembl** | **Genomicus** | **Symap** | **MizBee** |
| --- | --- | --- | --- | --- | --- |
| Open source | Y | Y | Available  on request | Y | Y |
| Genetic structural comparison | Y | N | N | N | N |
| Synteny / Gene order | Y | N | Y | Y | Y |
| Conserved genomic regions | N | Y | Y | Y | Y |
| Align sequences | Y | Y | N | Y | N |
| Export sequence and alignment | Y | Y | N | Y | N |
| Web based | Y | Y | Y | Y | N |
| Local | N | N | N | N | Y |

Table 1: Comparison of various phylogenetic visualisation tools with Aequatus.

Figure 1: The genetree for the monoamine oxidase (MAO) gene, with the Chimp gene as the reference, alongside other homologous genes in the exon-focused view.


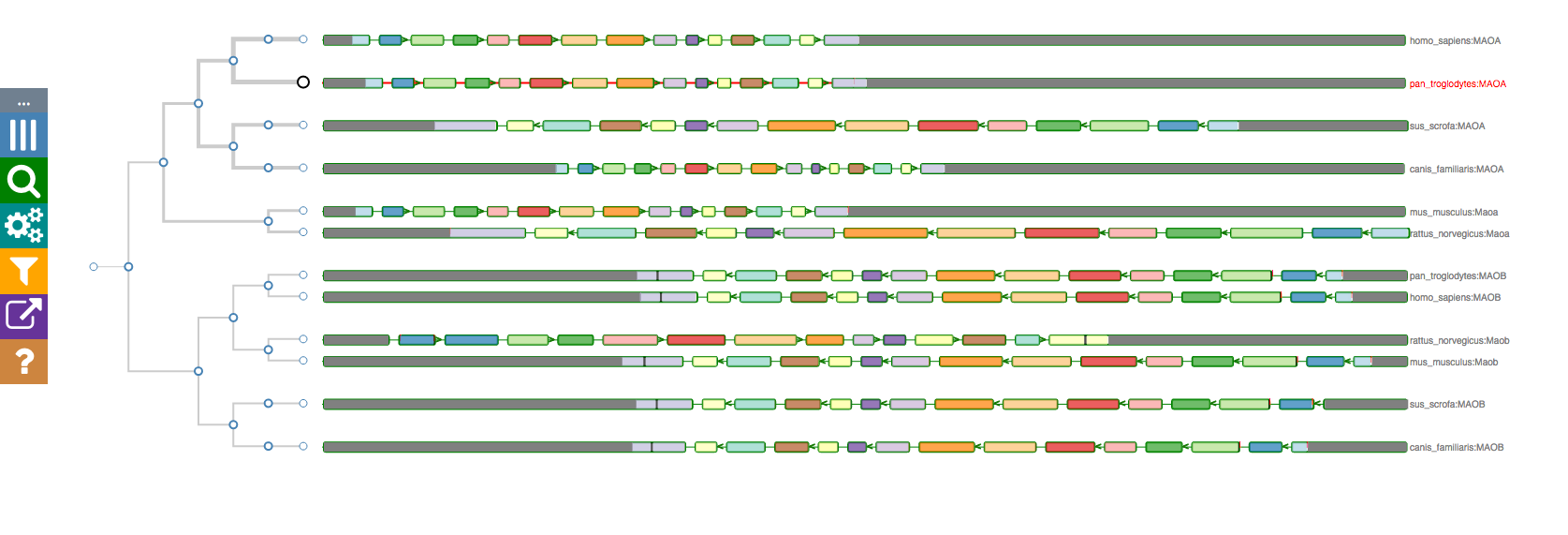

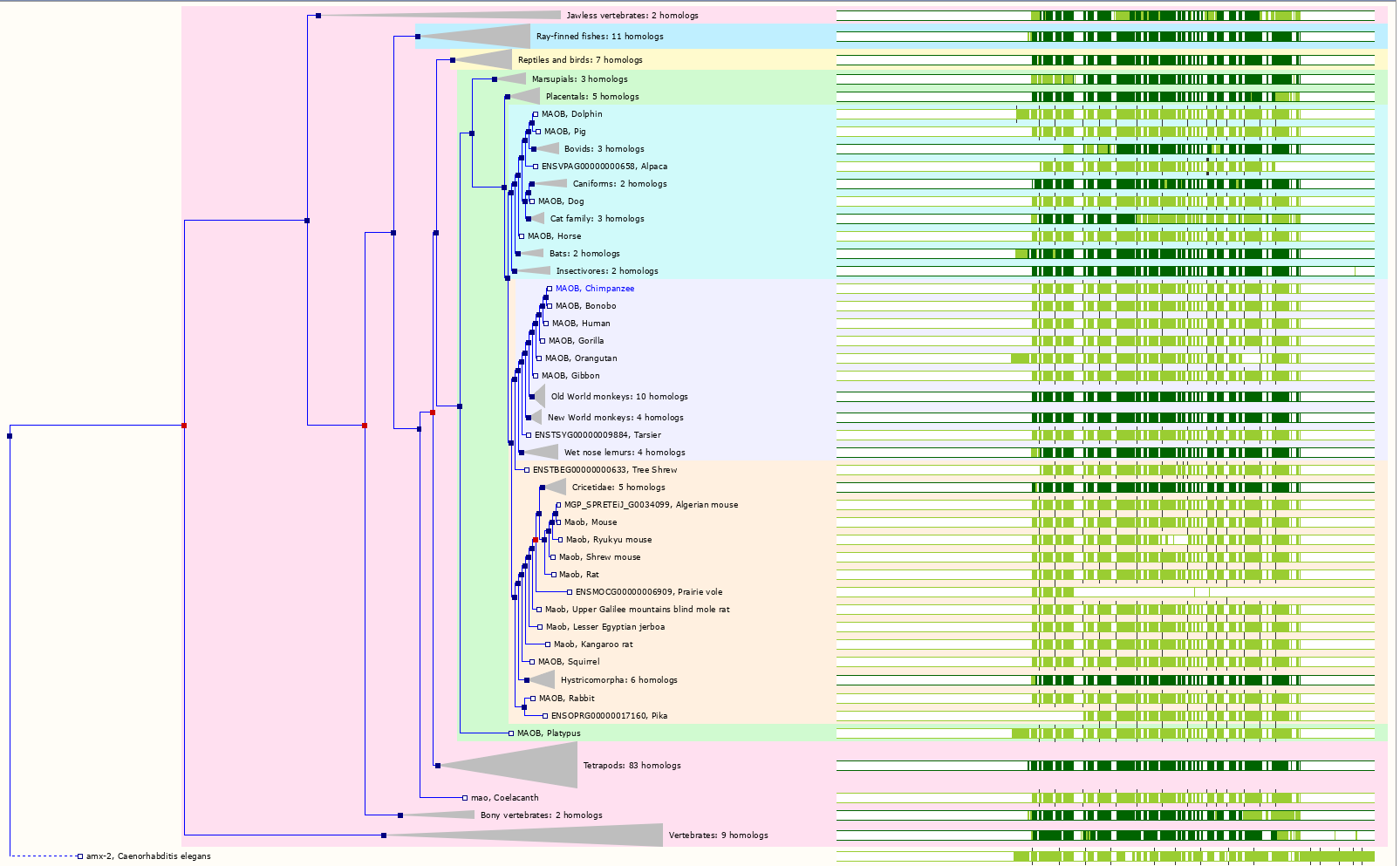


Figure 2: Ensembl visualising genetree for MAOB with the chimp gene as the reference, alongside other homologous genes along with alignments.


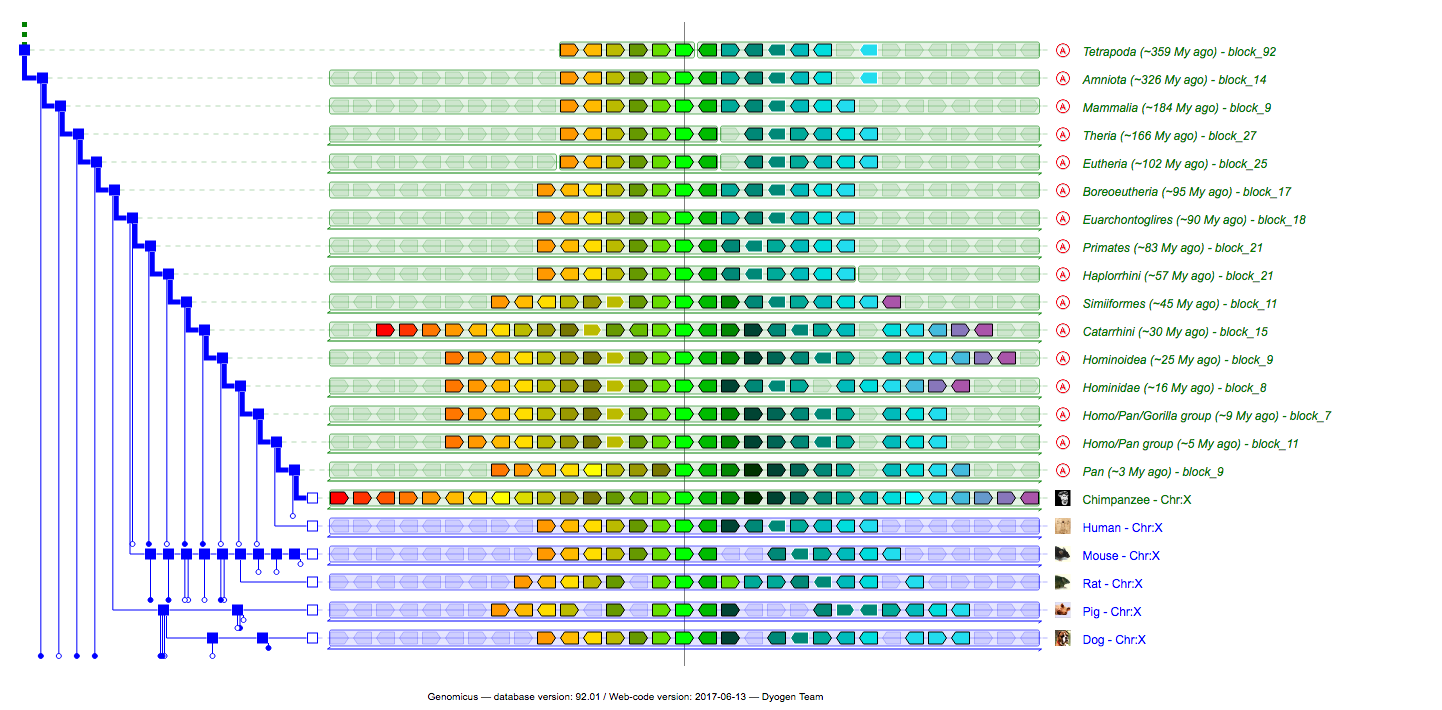


Figure 3: Genomicus visualising syntenic genes for MAOB with the chimp gene as the reference with neighbouring genes.
