# Supplementary material for: Aequatus: an open-source homology browser
Source: Gigascience. 2018 Nov 5;7(11):giy128. doi: 10.1093/gigascience/giy128 (PMC6251984; doi:10.1093/gigascience/giy128)
Supplement: Response_to_Reviewer_Comments_Original_Submission.pdf [file giy128_response_to_reviewer_comments_original_submission.pdf]

# Author's Response To Reviewer Comments

Close

Reviewer #1: The submission presents a web interface to compare gene structure across different closely related species. Starting with a reference gene, it provides the genomic neighbourhood and that of orthologous genes-thus enabling users to visualise local synteny (in a visualisation that is reminiscent of Genomicus). The key innovation, though, lies in a comparative display of gene structure, with an annotated gene tree on the left and a display of matching exons on the right. There are a few additional display options (tables, "sankay plots") but these are not nearly as important. Finally, there is an option to dynamically load information on the domain architecture of any gene.

The widget is well thought out and innovative, and so in general I think it will be quite useful to users. For resource developers, it is a real plus that Aequatus is available both with a full-fledged back-end integrated with Ensembl compara-which is a leading resource-while at the same time available as a JavaScript library only.

Inevitably I have a few points, but these should be easy to address in a revised manuscript.

Major point

1. From a user standpoint, where is or will Aequetus available? For instance, will http://aequatus.earlham.ac.uk/animal_compara/ be kept up to date with the latest Ensembl release? in any case, information about the release should be provided.

A. Yes, we will keep Aequatus available at the given web address. We will try our best to keep Aequatus updated with the latest release of Ensembl. We are also working on a new Aequatus version which will be able to retrieve data directly from Ensembl servers via its API. Moreover, we are going to add the Ensembl release version to the Aequatus webpage to keep the user informed.

2. From a developer standpoint, what is the rough scaling behaviour of the tool in terms of number of genome, number of genes per family etc? What are the bottlenecks? The point is that potential developers will want to know whether the widget can scale to their resource.

A. Aequatus shows the visualisation of a gene family reasonably fast, but since this is generated on the fly, it could behave slower with increasing size and complexity of the gene family (number of paralogues and number of genomes).

Minor points
3. It's not so clear what the colours mean in the gene structure view. I assume these are arbitrary colours that are nevertheless consistent across species, but I still wonder whether there is information in the fact that some exons have different intensities of the same hue, while others don't.

A. As pointed out by the referee the colour were arbitrarily selected to distinguish syntenic genes and matching exons respectively in the syntenic and gene tree views. We have now added a description in the Figure 2 legend.

4. Also unclear to me is the meaning of the tiny arrows between exons, as well as the very fine red, black and white lines. Consider adding a legend.

A. The arrows denote the strand on which the gene is located. We have also updated the Figure 3 legend in the manuscript, adding "In gene tree view, gray blocks at the start and end of each gene represent UTRs (untranslated regions), black bars within exons indicate insertions, red lines represent deletions specific to a given gene compared with the guide, and tiny arrows denotes the coding strand of the gene."

Discretionary/Suggestions:
5. It would be nice to have the possibility to save the current view as SVG, to facilitate inclusion in publications.

A. We appreciate the comment from reviewer about exporting current view as a SVG, this could be very useful. We are looking forward to integrate this into our next release.

Reviewer #2: Manuscript Title: Aequatus: An open-source homology browser
Manuscript Number: GIGA-D-18-00226
Reviewer: Deborah A Weighill

Summary
This study presents Aequatus, a web-based tool for the visualization of syntenic relationships as well as phylogenetic relationships between genes from pre-calculated alignments and gene features. Aequatus allows for the visualization of gene structure similarities in more detail than other tools, as it can visualize synteny at the exon level.

General Comments
This software will be a useful tool for the visualization of detailed syntenic relationships at the sub-gene level and at the localized gene level, though not at the large, chromosomal scale. The software meets the aims of the study to provide a tool to visualize both phylogenetic and syntenic information and the sub-gene level.

Code for this software is available on github and a demo web server is also available at links provided in the manuscript. The manuscript makes use of data from the Ensembl Compara and Core databases.

The language of this manuscript is of good quality.

Major Comments/Suggestions
1. The demo server appears to have a very limited number of species available for browsing. Is there a plan for this to be expanded so that this can be a browser for the entire Compara database?

A. We indeed plan to expand the set of species to host the Compara databases. The demo

server was intended to display Aequatus functionalities, but we are working on mirroring Ensembl Compara locally and then provide Aequatus running on it, serving all available species. We are also working on a new Aequatus version which will be able to retrieve data directly from Ensembl servers via its API to always make all species available at the latest release.

2. The authors list various other tools for synteny visualization and briefly mention how Aequatus differs from these tools. I think it is necessary to expand this discussion/comparison, for example, are these other tools webservers or tools to be run command line? Is there a major speed difference or differences in the size of input data that can be handled? A figure illustrating the output visualizations of these different tools, highlighting how Aequatus differs would be informative.

A. We have added a comparison table along with figures in the Supplementary material to compare the features of various phylogenetic visualisation tools with Aequatus. Regarding the comparison of performance: the performance of each tool is based on the technology used for development, the number of genomes and gene families visualised as well as the complexity of the data. Software performances will also be affected by the configuration of the host server and in some cases the local computer when the visualisation is generated locally. Thus, a performance comparison of various other tools will not enable us to establish a conclusion on which tool performs better.

3. The authors mention in the introduction that Aequatus "allows the identification of exon/intron boundary changes and mutations, informing the user about underlying genetic changes, but can also highlight mis-annotations, pseudogenes [17], or polyploidisation in animal and plant genomes." It is not explicitly illustrated in the manuscript how one can obtain this information using Aequatus. I think that an example (including figures) of how one can arrive at each of these biological insights mentioned in the introduction, using Aequatus, would be very useful. In addition, illustrating how Aequatus facilitates these interpretations better than other tools would be a good point to illustrate explicitly.

A. We have moved the cited sentence to the Discussion and clarified it, adding also an example figure where Aequatus is visualising a potential missannotation in the pig genome.

4. This paper appears to be companion paper to "GeneSeqToFamily: a Galaxy workflow to find gene families based on the Ensembl Compara GeneTrees pipeline" (Anil S Thanki et. al, https://doi.org/10.1093/gigascience/giy005). Citing this paper and explaining more how it fits into the Aequatus pipeline is necessary in my opinion.

A. We have now added more information about how the Aequatus visualisation tool is working together with the GeneSeqToFamily workflow in Galaxy.

Minor Comments/Suggestions
5. The manuscript abstract says that the software is available for download under an MIT license, whereas on the GitHub page it is distributed under the GNU General Public License. This should be made consistent.

A. We thank the reviewer for pointing this out. It was a mistake from our side as Aequatus.js is available under MIT license, whereas whole of Aequatus software is GPL v3 license, we have corrected this mistake in manuscript.

6. Captions for Figures 3 and 4 are missing colons after "Figure".

A. We have amended manuscript to correct this.

Close
