# Supplementary material for: Aequatus: an open-source homology browser
Source: Gigascience. 2018 Nov 5;7(11):giy128. doi: 10.1093/gigascience/giy128 (PMC6251984; doi:10.1093/gigascience/giy128)
Supplement: Reviewer_1_Report_(Original_Submission) -- Christophe Dessimoz — 7/17/2018 Reviewed [file giy128_reviewer_1_report_(original_submission).pdf]

Reviewer Report

Title: **Aequatus: An open-source homology browser**

Version: **Original Submission** **Date:** 7/17/2018

Reviewer name: **Christophe Dessimoz**

Reviewer Comments to Author:

The submission presents a web interface to compare gene structure across different closely related species. Starting with a reference gene, it provides the genomic neighbourhood and that of orthologous genes—thus enabling users to visualise local synteny (in a visualisation that is reminiscent of Genomicus). The key innovation, though, lies in a comparative display of gene structure, with an annotated gene tree on the left and a display of matching exons on the right. There are a few additional display options (tables, "sankay plots") but these are not nearly as important. Finally, there is an option to dynamically load information on the domain architecture of any gene.

The widget is well thought out and innovative, and so in general I think it will be quite useful to users. For resource developers, it is a real plus that Aequatus is available both with a full-fledged back-end integrated with Ensembl compara—which is a leading resource—while at the same time available as a javascript library only.

Inevitably I have a few points, but these should be easy to address in a revised manuscript.

Major point

- From a user standpoint, where is or will Aequetus available? For instance, will http://aequatus.earlham.ac.uk/animal_compara/ be kept up to date with the latest Ensembl release? in any case, information about the release should be provided.

- From a developer standpoint, what is the rough scaling behaviour of the tool in terms of number of genome, number of genes per family etc? What are the bottlenecks? The point is that potential developers will want to know whether the widget can scale to their resource.

Minor points
- It's not so clear what the colours mean in the gene structure view. I assume these are arbitrary colours that are nevertheless consistent across species, but I still wonder whether there is information in the fact that some exons have different intensities of the same hue, while others don't.

- Also unclear to me is the meaning of the tiny arrows between exons, as well as the very fine red, black and white lines. Consider adding a legend

Discretionary/Suggestions:
- It would be nice to have the possibility to save the current view as SVG, to facilitate inclusion in publications.

Methods

Are the methods appropriate to the aims of the study, are they well described, and are necessary controls included? Choose an item.

Conclusions

Are the conclusions adequately supported by the data shown? Choose an item.

Reporting Standards

Does the manuscript adhere to the journal's guidelines on minimum standards of reporting? Choose an item.

Choose an item.

Statistics

Are you able to assess all statistics in the manuscript, including the appropriateness of statistical tests used? Yes, and I have assessed the statistics in my report.

Quality of Written English

Please indicate the quality of language in the manuscript: Choose an item.

Declaration of Competing Interests

Please complete a declaration of competing interests, considering the following questions:

- Have you in the past five years received reimbursements, fees, funding, or salary from an organisation that may in any way gain or lose financially from the publication of this manuscript, either now or in the future?
- Do you hold any stocks or shares in an organisation that may in any way gain or lose financially from the publication of this manuscript, either now or in the future?
- Do you hold or are you currently applying for any patents relating to the content of the manuscript?
- Have you received reimbursements, fees, funding, or salary from an organization that holds or has applied for patents relating to the content of the manuscript?
- Do you have any other financial competing interests?
- Do you have any non-financial competing interests in relation to this paper?

If you can answer no to all of the above, write 'I declare that I have no competing interests' below. If your reply is yes to any, please give details below.

I declare that I have no competing interests

I agree to the open peer review policy of the journal. I understand that my name will be included on my report to the authors and, if the manuscript is accepted for publication, my named report including any attachments I upload will be posted on the website along with the authors' responses. I agree for my report to be made available under an Open Access Creative Commons CC-BY license (http://creativecommons.org/licenses/by/4.0/). I understand that any comments which I do not wish to be included in my named report can be included as confidential comments to the editors, which will not be published.

I agree to the open peer review policy of the journal

To further support our reviewers, we have joined with Publons, where you can gain additional credit to further highlight your hard work (see: https://publons.com/journal/530/gigascience). On publication of this paper, your review will be automatically added to Publons, you can then choose whether or not to claim your Publons credit. I understand this statement.

Yes Choose an item.
