# Supplementary material for: Aequatus: an open-source homology browser
Source: Gigascience. 2018 Nov 5;7(11):giy128. doi: 10.1093/gigascience/giy128 (PMC6251984; doi:10.1093/gigascience/giy128)
Supplement: Reviewer_2_Report_(Original_Submission) -- Deborah Weighill — 8/16/2018 Reviewed [file giy128_reviewer_2_report_(original_submission).pdf]

**Reviewer Report**

**Title: Aequatus: An open-source homology browser**

**Version: Original Submission** **Date:** 8/16/2018

**Reviewer name Deborah Weighill**

**Reviewer Comments to Author:**

Manuscript Title: Aequatus: An open-source homology browser
Manuscript Number: GIGA-D-18-00226
Reviewer: Deborah A Weighill

Summary
This study presents Aequatus, a web-based tool for the visualization of syntenic relationships as well as phylogenetic relationships between genes from pre-calculated alignments and gene features. Aequatus allows for the visualization of gene structure similarities in more detail than other tools, as it can visualize synteny at the exon level.

General Comments
This software will be a useful tool for the visualization of detailed syntenic relationships at the sub-gene level and at the localized gene level, though not at the large, chromosomal scale. The software meets the aims of the study to provide a tool to visualize both phylogenetic and syntenic information and the sub-gene level.

Code for this software is available on github and a demo web server is also available at links provided in the manuscript. The manuscript makes use of data from the Ensembl Compara and Core databases.

The language of this manuscript is of good quality.

Major Comments/Suggestions
1. The demo server appears to have a very limited number of species available for browsing. Is there a plan for this to be expanded so that this can be a browser for the entire Compara database?
2. The authors list various other tools for synteny visualization and briefly mention how Aequatus differs from these tools. I think it is necessary to expand this discussion/comparison, for example, are these other tools webservers or tools to be run command line? Is there a major speed difference or differences in the size of input data that can be handled? A figure illustrating the output visualizations of these different tools, highlighting how Aequatus differs would be informative.
3. The authors mention in the introduction that Aequatus "allows the identification of exon/intron boundary changes and mutations, informing the user about underlying genetic changes, but can also highlight mis-annotations, pseudogenes [17], or polyploidisation in animal and plant genomes." It is not explicitly illustrated in the manuscript how one can obtain this information using Aequatus. I think that an example (including figures) of how one can arrive at each of these biological insights mentioned in the introduction, using Aequatus, would be very useful. In addition, illustrating how Aequatus facilitates these interpretations better than other tools would be a good point to illustrate explicitly.
4. This paper appears to be companion paper to "GeneSeqToFamily: a Galaxy workflow to find gene families based on the Ensembl Compara GeneTrees pipeline" (Anil S Thanki et. al, https://doi.org/10.1093/gigascience/giy005). Citing this paper and explaining more how it fits into the Aequatus pipeline is necessary in my opinion.

Minor Comments/Suggestions
1. The manuscript abstract says that the software is available for download under an MIT license, whereas on the github page it is distributed under the GNU General Public License. This should be made consistent.
2. Captions for Figures 3 and 4 are missing colons after "Figure".

Recommendations
I think that this is a useful tool and that the manuscript should be published after addressing the suggested additional discussions above, particularly providing some more discussion of examples of biological conclusions to be drawn from such analyses, and a further discussion of the differences between this tool and other syntenic/phylogenetic visualization tools.

**Methods**

Are the methods appropriate to the aims of the study, are they well described, and are necessary controls included? Choose an item.

**Conclusions**

Are the conclusions adequately supported by the data shown? Choose an item.

**Reporting Standards**

Does the manuscript adhere to the journal's guidelines on minimum standards of reporting? Choose an item.

Choose an item.

**Statistics**

Are you able to assess all statistics in the manuscript, including the appropriateness of statistical tests used? Yes, and I have assessed the statistics in my report.

**Quality of Written English**

Please indicate the quality of language in the manuscript: Choose an item.

**Declaration of Competing Interests**

Please complete a declaration of competing interests, considering the following questions:

- Have you in the past five years received reimbursements, fees, funding, or salary from an organisation that may in any way gain or lose financially from the publication of this manuscript, either now or in the future?
- Do you hold any stocks or shares in an organisation that may in any way gain or lose financially from the publication of this manuscript, either now or in the future?

- Do you hold or are you currently applying for any patents relating to the content of the manuscript?
- Have you received reimbursements, fees, funding, or salary from an organization that holds or has applied for patents relating to the content of the manuscript?
- Do you have any other financial competing interests?
- Do you have any non-financial competing interests in relation to this paper?

If you can answer no to all of the above, write 'I declare that I have no competing interests' below. If your reply is yes to any, please give details below.

I declare that I have no competing interests

I agree to the open peer review policy of the journal. I understand that my name will be included on my report to the authors and, if the manuscript is accepted for publication, my named report including any attachments I upload will be posted on the website along with the authors' responses. I agree for my report to be made available under an Open Access Creative Commons CC-BY license (http://creativecommons.org/licenses/by/4.0/). I understand that any comments which I do not wish to be included in my named report can be included as confidential comments to the editors, which will not be published.

I agree to the open peer review policy of the journal

To further support our reviewers, we have joined with Publons, where you can gain additional credit to further highlight your hard work (see: https://publons.com/journal/530/gigascience). On publication of this paper, your review will be automatically added to Publons, you can then choose whether or not to claim your Publons credit. I understand this statement.

Yes Choose an item.
